# Effectiveness of nutritional support to improve treatment adherence in patients with tuberculosis: a systematic review

**DOI:** 10.1093/nutrit/nuad120

**Published:** 2023-09-27

**Authors:** Fasil Wagnew, Darren Gray, Tsheten Tsheten, Matthew Kelly, Archie C A Clements, Kefyalew Addis Alene

**Affiliations:** National Centre for Epidemiology and Population Health, College of Health and Medicine, The Australian National University, Canberra, Australian Capital Territory, Australia; College of Health Sciences, Debre Markos University, Debre Markos, Ethiopia; Population Health Program, QIMR Berghofer Medical Research Institute, Brisbane, Queensland, Australia; National Centre for Epidemiology and Population Health, College of Health and Medicine, The Australian National University, Canberra, Australian Capital Territory, Australia; National Centre for Epidemiology and Population Health, College of Health and Medicine, The Australian National University, Canberra, Australian Capital Territory, Australia; Penninsula Medical School, University of Plymouth, Plymouth, United Kingdom; Geospatial and Tuberculosis Research Team, Telethon Kids Institute, Nedlands, Western Australia, Australia; Faculty of Health Sciences, School of Population Health, Curtin University, Bentley, Western Australia, Australia

**Keywords:** nutritional support, treatment adherence, TB, systematic review

## Abstract

**Context:**

Nutritional interventions substantially improve tuberculosis (TB) treatment outcomes and prevent complications. However, there is limited evidence about the connections between having nutritional support and TB treatment adherence.

**Objective:**

The aim of this study was to determine the effectiveness of nutritional support in improving treatment adherence among patients with TB.

**Data Sources:**

Databases, including PubMed, Embase (Ovid), Web of Science, and Scopus, were comprehensively reviewed to identify relevant studies reporting the impacts of nutritional support on TB treatment adherence.

**Data Extraction:**

Two authors independently screened the title, abstracts, and full article texts to identify eligible studies and assess the risk of bias. Observational and interventional studies were included.

**Data Analysis:**

A narrative synthesis approach was used to summarize the findings qualitatively.

**Results:**

From the search, 3059 publications were identified; of these, 8 studies were included in this systematic review. Three types of nutritional interventions were identified: food baskets (eg, energy, micronutrient- or macronutrient-enriched food support), nutritional advice and guidance, and incentives for buying foods. Although 5 studies reported that nutritional support significantly improved treatment adherence in patients with TB, 3 studies showed that nutritional support had no effect on TB treatment adherence.

**Conclusions:**

Providing nutritional support may improve adherence to TB treatment. However, more well-powered, high-quality trials are warranted to demonstrate the effect of nutrition support on cost-effectively improving adherence to TB treatment.

**Systematic Review Registration:**

PROSPERO registration no. CRD42023392162.

## INTRODUCTION

Poor adherence to tuberculosis (TB) treatment is 1 of the major public health concerns that increases the risk of morbidity, mortality, and cost burden.^1^ According to reports from the United States, poor adherence to treatment is responsible for 125 000 deaths, 10% of hospital admissions, and $100 billion in healthcare costs every year.^2^^,^^3^ Although research has been conducted over the past few years to improve medication adherence rates, there has not been much change in treatment adherence.^4^^,^^5^ In particular, drug nonadherence is markedly higher among patients with chronic diseases as compared with those with acute diseases.^6^^,^^7^

Many patients with TB do not complete the full regimen necessary to recover from the disease. Previous studies estimated that the prevalence of poor adherence to TB treatment ranged from 33% to 50%.^8^^,^^9^ This is a significant contributor to prolonged transmission of TB, amplification of drug resistance, hampered treatment success, and catastrophic economic effects, especially in the presence of comorbidities.^10^^,^^11^ A recent meta-analysis also reported that missing more than 10% of doses of the prescribed TB drugs causes a 6-fold higher risk of poor treatment outcomes.^12^

There are multiple factors that can be associated with poor adherence to TB treatment, of which3 are major factors: patient status, healthcare delivery systems, and healthcare providers.^13–16^ Nutrition-related factors such as malnutrition and a weakened immune system are reported as major contributors of poor treatment adherence.^17^^,^^18^ In addition, household food insecurity is a significant contributor to TB treatment nonadherence in low- and middle-income countries.^19^^,^^20^ It can be challenging for people with food insecurity to complete their TB treatment for an extended time (ie, a minimum of 6 months). A qualitative study in Swaziland showed that most patients with food insecurity discontinued their treatment because it increased their appetite and they already had insufficient access to food.^21^ The presence of food insecurity leading to undernutrition is also associated with amplified disease severity, compromised treatment outcomes, and heightened mortality rates.^17^^,^^22^ Thus, interventions focusing on food security could improve adherence to TB treatment.^23^^,^^24^ Providing nutritional support to patients and their families is a vital motivator that enhances adherence to treatment plans and serves as a safeguard against the potentially overwhelming financial burden caused by TB.^25^

A comprehensive intervention aiming to maximize adherence to TB treatment is needed to prevent treatment interruption and the transmission of the disease in the community.^26^ Directly observed treatment, short-course (DOTS), has been 1 of the most commonly used interventions since 1993^27^ to increase adherence to TB treatment, despite that it requires further enablers such as financial and food support.^28^ Accordingly, a variety of public health program strategies, including financial incentives, nutrition support, and digital technologies, have also been implemented to improve adherence to TB drugs.^29^ Understanding the effectiveness of these strategies in improving adherence to TB treatment merits special consideration.

Previous studies have provided evidence that nutritional intervention can improve treatment outcomes and its prognostic markers among patients with TB.^30–32^ However, evidence about the potential of nutritional support to improve adherence to TB treatment remains limited and inconclusive.^23^ Therefore, in this systematic review, we aimed to examine the effectiveness of nutritional support to improve treatment adherence in patients with TB.

## METHODS

This systematic review was developed on the basis of the Preferred Reporting Items for Systematic Reviews and Meta-analysis (PRISMA) recommendations^33^ and was registered with the International Prospective Register of Systematic Reviews (PROSPERO; identifier CRD42023392162). The PRISMA checklist is provided in Table S1 in the Supporting Information online.

### Study selection and eligibility criteria

Observational and interventional studies were included according to the research questions developed using the PICOS (Population, Intervention, Comparator, Outcomes, and Studies) format^34^ (Table 1). Conference and meeting abstracts without adequate information, articles in languages other than English, animal studies, systematic reviews, and those with insufficient information on the primary outcomes of interest were excluded.

**Table 1:** PICOS criteria for inclusion of studies

Parameter	Inclusion criteria
Population	Patients with pulmonary tuberculosis aged ≥15 y, based on the World Health Organization classification of adults,^61^ with acid-fast bacilli sputum smear-positive or smear-negative, with or without comorbidities, were considered.
Intervention	Additional food support, macronutrient or micronutrient supplementation, incentives for groceries, and nutritional guidance or advice, which seeks to improve or assist treatment adherence
Comparator	Patients with tuberculosis who did not receive nutritional support
Outcomes	Studies on nutritional support targeted at the improvement of adherence to tuberculosis treatment
Study design	Randomized controlled trials, quasi-experimental, cohort, case–control, and cross-sectional studies

### Search strategy

A comprehensive search was undertaken in the PubMed, Embase (via Ovid), Web of Science, and Scopus databases for relevant studies published between January 1, 2000, and January 1, 2023. Grey literature and reference lists of identified articles were hand-searched for additional relevant studies missed in the initial search strategy.

The search strategy combined key terms such as “tuberculosis,” “nutritional intervention,” “food support,” “treatment adherence,” and “treatment compliance.” The full search strategies for each database are provided in the Table S2 in the Supporting Information online. Searches were conducted between December 2, 2022 to January 30, 2023.

### Risk of bias

Risk of bias was evaluated using 2 published quality-rating scales: the Cochrane risk of bias 2 (RoB 2)^35^ for randomized controlled trials (RCTs) and the Risk of Bias in Nonrandomized Studies of Interventions (ROBINS-I) tool^36^ for non-RCTs. Two authors (F.W. and T.T.) independently evaluated the risk of bias for each included article. When there was any disagreement, the 2 authors reached a decision by consensus. Studies were categorized as at low risk of bias when all important contents were evaluated and found to be at low risk. A risk of bias was assessed what the authors reported having done for each domain in each study and then made a decision as to whether the study as “low,” “high,” or “unclear” risk of bias. The risk of bias assessment tools are listed in Table S3 in the Supporting Information online.

### Data extraction and summarization

After removing duplicate articles from the Endnote 20 software library, 2 authors (F.W. and T.T.) independently screened the title, abstract, and full text of each article to identify eligible studies and extracted the required information from the included articles using a standardized Joanna Briggs Institute data extraction form. Discrepancies were resolved through discussion. Data on primary author, year of publication, country of study, study period, study design, sample size, types of nutritional interventions, duration of follow-up, and outcomes were extracted by the reviewers using a standardized data extraction format. Because of the presence of a high degree of heterogeneity across the included studies, a meta-analysis was not performed. Instead, a narrative synthesis was used to qualitatively summarize the effect of nutritional support on TB treatment adherence.

## RESULTS

**Figure 1:**
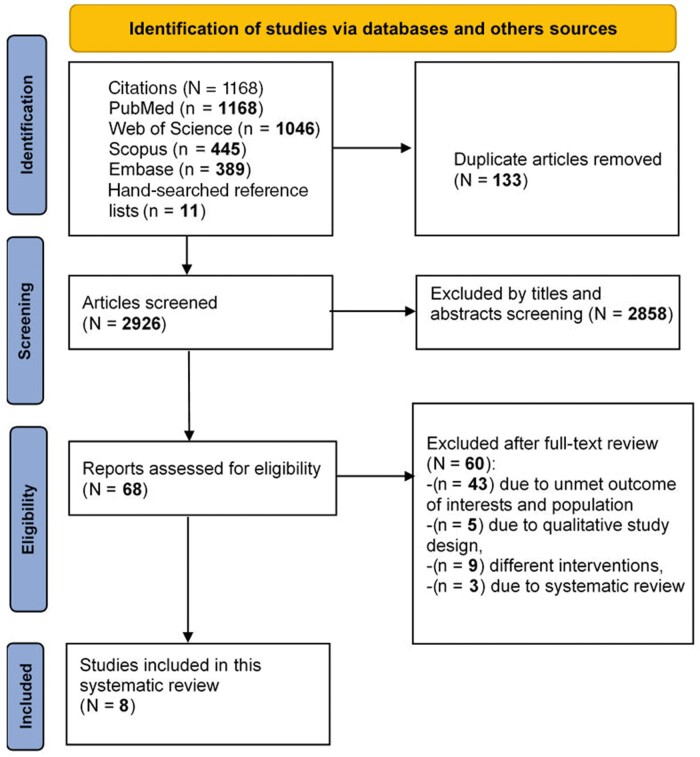
Flow diagram of the literature search process.

Initially, 3048 articles were obtained from the electronic databases search and 11 additional articles were found in the hand-search of reference lists of the included studies. After the removal of duplicates, a total of 2926 records were screened for title and abstract, which resulted in 68 articles for full-text reviewing. Finally, in the full-text review, 5 interventional studies^37–41^ and 3^42–44^ retrospective comparative studies met the inclusion criteria, comprising 1467 participants. A flowchart showing the selection process of articles is provided in Figure 1.

### Characteristics of the included studies

The characteristics of the 8 included studies are provided in Table 2.^37–44^ All included studies were conducted in low- and middle-income countries; 4 studies were conducted in the upper-middle-income countries of China,^38^ Russia,^44^ Georgia,^42^ and Brazil^43^; and 4 were conducted in the lower-middle-income countries of Senegal,^37^ Tanzania,^39^ India,^41^ and Timor-Leste (East Timor).^40^ The included studies focused on the most vulnerable groups of population. For instance, 3 studies^37^^,^^39^^,^^41^ were conducted with patients with TB and human immunodeficiency virus (HIV) coinfection, and 3^40^^,^^42^^,^^44^ were conducted with a high proportion of homeless or malnourished adults. Only 2 studies^38^^,^^43^ involved members of the general adult population with TB.

**Table 2:** Characteristics and summary description of included studies in assessing the effectiveness of nutritional support to improve treatment adherence in patients with tuberculosis

Reference	Country	Study design and period	Participants, N	Intervention group	Comparator group	Follow-up period	Outcomes of interest and main findings
Martins et al, 2009^40^	Timor-Leste (East Timor)	RCT November–July 2006	270	Patients received additional food every time they attended the clinic. In the intensive phase, each day they were provided at the clinic 1 bowl of feijuada, meat, red kidney beans, and vegetable stew with rice. In the continued phase, patients received unprepared food to take home (red kidney beans, rice, and oil) (1 meal/d per adult).	Patients received nutritional advice.	8 mo	Primary outcome included the completion of treatment. Secondary outcomes included adherence to treatment, weight gain, and clearance of sputum smears. Findings: In this setting food supplementation did not significantly improve treatment adherence or treatment outcome.
Benzekri et al, 2019^37^	Senegal	RCT June–August 2017	26	Patients received a monthly food basket consisting of 5 kg of cowpeas (*Vigna unguiculata*) and 3 kg of rice grown in Senegal, 1.5 L of vegetable oil, and 1.6 kg of powdered milk.	Patients received RUTF.	6 mo	Primary outcome included the treatment adherence. No significant effect has been found; medication adherence for 7 d RUTF (96.8%; food basket, 95.1%) and week 4 of RUTF (98%; food basket, 98.4%).
Hu et al, 2021^38^	China	Interventional studies October 2019–October 2020	123	Patients received health education and dietary guidance about nutritious foods, vitamins, and avoiding alcohol use.	Patients in the comparator arm received regular care and food advice.	6 mo	Findings: The treatment adherence rate of patients with TB in the intervention group (96.83%) was significantly higher compared with the control group (75%).
Filho et al, 2009^43^	Brazil	Historical comparative study September 2001–July 2006	142	Patients received baskets of nonperishable food, distributed monthly.	Patients received only the standard TB treatment care.	At the end of TB treatment	TB treatment outcome was a primary outcome of interest. Findings: The statistical comparison between the 2 groups revealed that the rate of poor adherence was markedly lower in the intervention group (12.9% vs 30.3%).
Sudarsanam et al, 2011^41^	India	RCT January–November 2005	103	Patients received a locally prepared cereal–lentil mixture providing 930 kcal and a multivitamin micronutrient supplement.	Patients in the comparator arm received only standard TB drugs.	After TB treatment end	The primary outcome of interest was TB treatment outcomes. Secondary outcomes were body composition, adherence and condition on follow-up 1 y after cessation of TB therapy and supplementation. Findings: The overall adherence to TB treatment was not statistically significance between the 2 groups(*P* = 0.197).
Gärden et al, 2013^44^	Russia	Historical comparative study December 2001–January 2004	Intervention: 142 Control: 376	Patients received food packages once a day 5 d/wk, which comprised canned meat, bread, butter, egg, soup with cream, juice, tea, and yogurt.	Patients received only standard TB drugs	6 mo	The primary outcome of interest was adherence to TB treatment. Findings: Patients receiving food supports had a good treatment adherence
Jeremiah et al, 2014^39^	Tanzania	RCT September 2010–August 2011	100	Patients received a nutritional supplement in the form of high-energy and vitamin/mineral-enriched biscuits for 2 mo.	Patients received only standard TB treatments.	After 2 mo	Primary outcome was rifampicin exposure. Adherence to TB treatment was also reported. Poor treatment adherence was higher among patients who were not receiving nutritional supports (3.9% vs 12.3%).
Bock et al, 2001^42^	Georgia	Historical comparative study November 1996–October 1997	185	Patients received a $5 grocery coupon for each DOT appointment.	Patients received only standard TB treatments.	At the end of TB treatment	There was a strong association between the use of incentives and increased adherence.

*Abbreviations:* DOT, directly observed treatment; RCT, randomized, controlled trial; RUTF, ready-to-use therapeutic food; TB, tuberculosis.

All included studies were published between 2001 and 2021. Studies varied in sample size from 26 participants in Senegal^37^ to 518 participants in Russia.^44^ Five^37–39^^,41,43^ of the 8 studies (62.5%) were underpowered; they included fewer than 75 participants per arm (Table 2).

### Intervention types and compositions

The included studies used a variety of nutritional interventions. Six studies used interventions combining high-energy food baskets and micronutrient supplementations,^37^,^39–41^^,^^43^^,^^44^ comprising varieties of meals, including meat, powdered milk, red kidney beans, rice, locally prepared cereals, and vitamin- or mineral-enriched biscuits.

A study in China^38^ used interventions combining dietary guidance, which advised patients to consume high-protein foods and vitamin B–enriched foods, and avoid smoking and alcoholic drinks. Last, Bock et al^42^ used incentives for groceries to improve TB treatment adherence.

Of the 8 included studies, 5 used clinic-based DOTS to assess treatment adherence,^40–44^ and 2 studies relied on patient self-reporting.^37^^,^^38^ Only 1 study did not provide details on the methods used for measuring treatment adherence.^39^

### Effectiveness of nutritional support on tuberculosis treatment adherence

Two studies^38^^,^^42^ concluded that the proportion of patients with good treatment adherence in the intervention group was significantly higher than that of the comparator group. In addition, 3 studies reported that the proportion of poor adherence was lower in the intervention group than in the comparator group: 12.9% vs 30.3% in Brazil,^43^ 27% v 67% in Russia,^44^ and 3.9% vs 12.3% in Tanzania.^39^

However, 3 other studies^37^^,^^40^^,^^41^ concluded that treatment adherence was not significantly associated with the nutritional support compared with those who did not receive the nutritional support. Of those, 2 studies^37^^,^^40^ did not have clear control and intervention groups. Both groups received nutritional interventions: the intervention group received food baskets, and the control group received nutritional advice. Studies in Senegal^37^ and India^41^ included only patients with TB and HIV coinfection.

There were substantive heterogeneities in study settings, types of interventions, and adherence measures among the included studies, and the data were not similar enough to combine different studies. Therefore, there was insufficient common ground for estimating pooled effect sizes using meta-analysis.

### Assessment of risk of bias

Assessment of methodological quality and risk of bias in the RCTs reviewed is shown in Table S3 in the Supporting Information online. All trials reported sufficient details of random sequence generation and a low selection bias was found because all publications of RCTs provided information about the processes of random sequence generation in the studies. Blinding to participant’s performance bias was high for 2 trials,^40^^,^^41^ low for 1 trial,^37^ and unclear for the remaining trial.^39^ Overall, there was low attrition bias (incomplete outcome data) across the trials because all trials reported a low proportion of dropout rates (see Table S3 in the Supporting Information online).

Risk-of-bias assessment for non-RCTs is presented in Table S3 in the Supporting Information online. Bias due to confounding was high across studies except that of Hu et al.^38^ Measurement of outcome bias was high across the studies because the evaluators were aware of interventions. Studies varied with respect to attrition bias; risk of attrition bias was low in 2 studies,^38^^,^^43^ whereas that of the remaining 2 studies^42^^,^^44^ was unclear. The included studies were unclear regarding reporting bias because the protocols were unavailable online (see Table S3 in the Supporting Information online). Further details of the quality assessment for the RCTs and non-RCTs are provided in the Table S3 in the Supporting Information online.

## DISCUSSION

Given the paucity of evidence of interventions improving treatment adherence, we aimed in this systematic review to examine the recent literature focusing on nutritional support strategies that have been determined to enhance treatment adherence among patients with TB. Eight studies comprising a total of 1467 participants were included. Five of the 8 studies showed that nutritional support was associated with improved adherence to TB treatment. However, the remaining 3 studies showed no significant effect on improving adherence to TB treatment.

Globally, enhancing adherence to TB treatment and then getting a successful treatment outcome are vital public health goals. Accordingly, greater than 90% treatment adherence is a target for controlling the spread and preventing drug-resistant TB. This is warranted for successful treatment outcomes and a key element to achieving the World Health Organization’s End TB targets.^45^ However, evidence indicates that many patients with TB discontinue treatment during the follow-up period. Adherence is a complicated phenomenon that can be associated with multiple factors at the individual and institutional levels.^46^ Even achieving the global End TB goal will be challenging if nonadherence is left unaddressed. The DOTS strategy has long been helpfully implemented in many countries. However, additional measures might be required, including financial and food support.^47^^,^^48^

In this review of the nutritional supports described in 8 studies, 6 studies focused on food baskets highly enriched with macro- or micronutrients, 1 on nutritional advice and guidance, and 1 on incentives for buying groceries. Accordingly, 5 studies reported significantly improved treatment adherence among patients receiving support compared with patients without nutritional support, as evidenced by an increased proportion of adherence or markedly decreased nonadherence. This finding is consistent with a previous review of patients with TB and HIV coinfection, which found improved adherence to treatment among those who received food support.^23^ Similar studies among patients living with HIV found a positive relationship between food support and adherence to antiretroviral therapy.^49^^,^^50^ In addition, a systematic review of qualitative studies illustrated that a food shortage was identified as a common barrier to treatment adherence for patients living with TB.^24^ This could be because inadequate nutrition support is likely to increase undernutrition and the adverse effects of TB treatment,^19^ which, in turn, hamper adherence to and completion of care and treatment. Therefore, it is important to note that food support has multiple roles: it is an enabler to initiate and continue TB treatment, contributes to managing malnutrition,^51^ and mitigates the social and financial burdens of TB at the individual and household levels.^52^

In contrast, 3^37^^,^^40^^,^^41^ of the 8 studies reported no significant association with improved adherence to TB treatment when nutritional intervention was added to the standard TB treatments. This nonsignificant therapeutic effect might be due to various factors. For instance, more than 60% of all included studies had inadequate sample sizes, which may have resulted in missing a meaningful difference in treatment adherence between groups even when improving treatment adherence substantially. Most importantly, 2 of 3 studies^37^^,^^40^ did not have clear control and intervention groups, because both groups received nutritional interventions. Also, the 2 included studies^37^^,^^41^ with a nonsignificant effect on adherence were carried out among patients with TB and HIV coinfection that may need special consideration. For instance, in previous trials examining the effect of nutritional interventions on TB treatment outcomes among patients with TB and HIV coinfection, no significant effect was found.^53–55^ This may indicate that the nutritional intervention in patients with TB and HIV coinfection may need an extra dose to be effective, owing to malabsorption and increased utilization of nutrients.^56^ Patients with TB and HIV coinfection may also need close follow-up because they may have been sicker and less able to take their prescribed medications and nutritional interventions.

Furthermore, marked heterogeneity in the composition, form, and amount of nutritional interventions across the countries also contributed to these observed variabilities. A meta-analysis was not performed, due to the presence of high heterogeneity between the studies. Therefore, future clinical trials would be needed to confirm these findings.

Most recently, food support is mentioned in the World Health Organization TB treatment guidelines,^20^ but it is not well explained in the context of improving adherence to TB treatment. In addition, material incentives, in combination with other forms of social support, are thought to improve adherence and treatment outcomes for various diseases by directly influencing patients’ health behaviours.^57^^,^^58^ This approach is also essential for patients with TB because the disease mainly affects poor people. Patient-centered support interventions such as enablers and food incentives should be incorporated into the current TB control program to help patients overcome some of the economic constraints affecting their treatment adherence.^8^ For instance, the World Health Organization developed a post-2015 Global TB strategy that explicitly encompasses the role of universal health coverage and social protection, including food support.^59^ The Government of India has also announced a monthly direct benefit transfer for patients with TB to enable the purchase of high-protein foods.^60^ Therefore, integrated interventional strategies have to be cost-effectively tailored to the clinical practice; otherwise, benefits are substantially reduced in usual clinical practice with low adherence rates. Accordingly, standardized nutritional supports may provide enormous benefits to improve TB treatment adherence in areas with a high prevalence of food insecurity and undernutrition.

### Implications for future research

Considering the growing global risk of drug-resistant TB, it is crucial to prioritize efforts aimed at assisting patients to successfully complete their TB treatment. Therefore, high-quality, well-powered, and multicentric clinical trials of nutritional support on improving adherence to TB treatment are urgently warranted to confirm this finding. Such trials should ideally stratify by comorbid status (eg, HIV) to examine differential dose-response effects in these key, high-risk populations. Implementation research will also be relevant to evaluate the feasibility, acceptability, and cost-effectiveness of the various form of nutritional support in diverse communities and various geographical areas.

### Limitations of the study

This systematic review has some important limitations that must be kept in mind when interpreting the findings. Despite an extensive search, we only found 8 relevant articles. The majority of the included studies had a limited sample size, which could potentially compromise the precision of these findings. As well, only articles published in the English language were included, which may miss relevant articles not published in English. In addition, some studies used patient self-report to measure treatment adherence in the context of clinical care. However, this method may overestimate adherence score as compared with objective adherence measures such as electronic drug prescriptions and laboratory tools. Wide heterogeneities in the description of interventions and outcome evaluations were observed across the studies, so a meta-analysis and a stratified analysis were not performed. Furthermore, this systematic review did not provide a detailed review of studies that highlighted exclusively on cost, feasibility, and acceptability of the interventions.

## CONCLUSION

Providing nutritional support may improve adherence to TB treatment. However, more research with adequate power is warranted to demonstrate the effect of nutritional support on cost-effectively improving adherence to TB treatment. The body of research supporting nutritional supports designed to increase TB treatment adherence is incompletely understood, with 5 studies reporting improved adherence to TB medications in the intervention group, and 3 reporting no association between the 2 groups.

## Supplementary Material

nuad120_Supplementary_Data

## Data Availability

All data generated or analyzed during this study are included in this published article.

## Supporting Information

The following Supporting Information is available through the online version of this article at the publisher’s website.

*Table S1*  **PRISMA 2020 checklist**

*Table S2* **Systematic review search terms** in PubMed, Scopus, Embase, and Web of Science **(December 25, 2022)**

*Table S3* **Assessment of bias of RCTs included in the systematic review using RoB2**
