# Supplementary material for: Effectiveness of nutritional support to improve treatment adherence in patients with tuberculosis: a systematic review
Source: Nutr Rev. 2023 Sep 27;82(9):1216–25. doi: 10.1093/nutrit/nuad120 (PMC11317773; doi:10.1093/nutrit/nuad120)
Supplement: nuad120_Supplementary_Data [file nuad120_supplementary_data.zip › nuad120_Supplementary_Data/PRISMA_2020_checklist.docx]

| **Section and Topic** | **Item #** | **Checklist item** | **Location where item is reported** |
| --- | --- | --- | --- |
| **TITLE** | | |  |
| Title | 1 | Effectiveness of nutritional support to improve treatment adherence in people with tuberculosis: a systematic review | 2 |
| **ABSTRACT** | | |  |
| Abstract | 2 | Abstract  Introduction: Nutritional interventions substantially improve tuberculosis (TB) treatment outcomes and prevent complications. However, there is limited evidence about the connections between having nutritional support and TB treatment adherence. This study thus aimed to determine the effectiveness of nutritional support in improving treatment adherence among people with TB.  Methods: We searched PubMed, Embase (Ovid), Web of Science, and Scopus for relevant studies reporting the impacts of nutritional support on TB treatment adherence. Two authors independently screened the title, abstracts, and full text to identify eligible studies and assessed the risk of bias. A narrative review was undertaken to qualitatively summarise the findings.  Findings: From the search, we identified 3059 publications; of these, eight studies were included in this systematic review. Three types of nutritional interventions were identified, which include food baskets (eg; energy, micronutrient or macronutrient enriched food support), nutritional advice and guidance, and incentives for buying foods. While five studies reported that nutritional support significantly improved treatment adherence in people with TB, three studies showed that nutritional support had no effect on TB treatment adherence.  Interpretations: Providing nutritional support may improve adherence to TB treatment. However, further well-powered, high-quality trials are warranted to demonstrate the effect of nutrition support on cost-effectively improving adherence to TB treatment. | 3 |
| **INTRODUCTION** | | |  |
| Rationale | 3 | Previous studies have provided evidence that nutritional intervention can improve treatment outcomes and its prognostic markers among people with TB. However, evidence about the potential of nutritional support to improve adherence to TB treatment remains limited and inconclusive. | 5 |
| Objectives | 4 | Therefore, this systematic review aimed to examine the effectiveness of nutritional support to improve treatment adherence in people with TB. | 5 |
| **METHODS** | | |  |
| Eligibility criteria | 5 | People with pulmonary TB aged 15 and older —based on the WHO classification of adults— with acid-fast bacilli (AFB) sputum smear-positive or smear-negative, with or without comorbidities, were considered. Conference and meeting abstracts and articles in languages other than English, animal studies, systematic reviews, and those with insufficient information on the primary outcomes of interest were excluded. | 6 |
| Information sources | 6 | A comprehensive search was undertaken in PubMed, Embase (via Ovid), Web of Science, and Scopus for relevant studies that had been published between 1 January 2000 and 1 January 2023. Grey literature and reference lists of identified articles were hand-searched for additional relevant studies missed in the initial search strategy. | 6 |
| Search strategy | 7 | The search strategy combined key terms such as “tuberculosis”, “nutritional intervention”, “food support, “treatment adherence”, and “treatment compliance”. The full search strategies for each database are provided in the supplementary material (Supplementary file 1). | 6 |
| Selection process | 8 | After removing duplicate articles from Endnote 20 software library, two authors (FW and TT) independently screened the title, abstract and full text to identify eligible studies and extracted the required information from the included articles using a standardised Joanna Briggs Institute (JBI) data extraction form. | 6 |
| Data collection process | 9 | Two authors (FW and TT) independently screened the title, abstract and full text to identify eligible studies and extracted the required information from the included articles. | 6 |
| Data items | 10 | Data on primary author, year of publication, country of study, study period, study design and sample size, types of nutritional interventions, duration of follow-up, and outcomes were extracted. | 6 |
| Study risk of bias assessment | 11 | Risk of bias was evaluated using two published quality-rating scales: the Cochrane risk-of-bias 2 (RoB 2) for RCTs and the risk of bias in non-randomised studies of interventions tool (ROBINS-I) for non-RCTs. | 6 |
| Effect measures | 12 | Due to the presence of a high degree of heterogeneity across the included studies, we did not perform a meta-analysis. Instead, a narrative synthesis was used to qualitatively summarise the effect of nutritional support on TB treatment adherence. | 6-7 |
| Synthesis methods | 13a | The characteristics of the included articles were summarized descriptively in tables. | 6-7 |
|  | 13b | Instead, a narrative synthesis was used to qualitatively summarise the effect of nutritional support on TB treatment adherence. | 6-7 |
|  | 13d | - | - |
|  | 13e | - | - |
| Reporting bias assessment | 14 | Risk of bias was evaluated using two published quality-rating scales: the Cochrane risk-of-bias 2 (RoB 2) for RCTs and the risk of bias in non-randomised studies of interventions tool (ROBINS-I) for non-RCTs. | 6 |
| Certainty assessment | 15 | - | - |
| **RESULTS** | | |  |
| Study selection | 16a | We obtained 3048 articles from our electronic databases search and 11 additional articles from a hand-search of reference lists of the included studies. After the removal of duplicates, a total of 2926 records were screened for title and abstract, which resulted in 68 articles for full-text reviewing. Finally, in the full-text review, five interventional studies and three retrospective comparative studies met our inclusion criteria, comprising 1467 participants | 7 |
|  | 16b | - | - |
| Study characteristics | 17 | Finally, in the full-text review, five interventional studies and three retrospective comparative studies met our inclusion criteria, comprising 1467 participants | 7 |
| Risk of bias in studies | 18 | Assessment of methodological quality and risk of bias in the RCTs reviewed is shown in table S1 &S2. | 8 |
| Results of individual studies | 19 | The included studies result are presented in Table 1. | Table 1 |
| Results of syntheses | 20a | The included studies result are presented in Table 1. | 8 |
|  | 20b | Five out of eight studies showed that nutritional support was associated with improved adherence to TB treatment. However, the remaining three studies showed no significant effect on improving adherence to TB treatment. | 7 |
|  | 20c | We did not perform meta-analysis and sub-group analysis. | - |
| **DISCUSSION** | | |  |
| Discussion | 23a | Given the paucity of evidence on interventions improving treatment adherence, this systematic review aimed to examine the recent literature focussing on nutritional support strategies that have been determined to enhance treatment adherence among people with TB. We included eight studies comprising a total of 1467 participants from six LMICs. Five out of eight studies showed that nutritional support was associated with improved adherence to TB treatment. However, the remaining three studies showed no significant effect on improving adherence to TB treatment. | 9 |
|  | 23b | Despite an extensive search, we only found eight relevant articles. As well, we solely included articles published in English language, which may miss relevant non-English articles. In addition, some studies used patient self-report to measure treatment adherence in the context of clinical care. | 11 |
|  | 23c | We solely included articles published in English language, which may miss relevant non-English articles. | 11 |
|  | 23d | Providing nutritional support may improve adherence to TB treatment. However, further research with adequate power is warranted to demonstrate the effect of nutritional support on cost-effectively improving adherence to TB treatment.  The body of research supporting nutritional supports designed to increase TB treatment adherence is incompletely understood, with five studies reported improved adherence to TB medications in the intervention group, while three reported no association between the two groups. | 12 |
| **OTHER INFORMATION** | | |  |
| Registration and protocol | 24a | This was registered with the International Prospective Register of Systematic Reviews (PROSPERO: CRD42023392162). | 5 |
| Support | 25 | None declared | 12 |
| Competing interests | 26 | None declared | 12 |
| Availability of data, code and other materials | 27 | All data generated or analysed during this study are included in this published article. | 12 |

***Reference***

*S1:* Page MJ, McKenzie JE, Bossuyt PM, Boutron I, Hoffmann TC, Mulrow CD, et al. The PRISMA 2020 statement: an updated guideline for reporting systematic reviews. BMJ 2021;372:n71. doi: 10.1136/bmj.n71

For more information, visit: <http://www.prisma-statement.org/>
