# Supplementary material for: Effectiveness of nutritional support to improve treatment adherence in patients with tuberculosis: a systematic review
Source: Nutr Rev. 2023 Sep 27;82(9):1216–25. doi: 10.1093/nutrit/nuad120 (PMC11317773; doi:10.1093/nutrit/nuad120)
Supplement: nuad120_Supplementary_Data [file nuad120_supplementary_data.zip › nuad120_Supplementary_Data/Suplementary file 1 search strategies.docx]

**Systematic review search terms in PubMed, Scopus, Embase and Web of Science (searching time December 25, 2022)**

1. **Searching in PubMed searching**

| **No** | **Search terms** | **Results** |
| --- | --- | --- |
| ≠4 | ≠1 and ≠2 and ≠3 | 1,168 |
| ≠3 | ((((((((((((("Treatment adherence") OR ("Therapeutic Adherence and Compliance")) OR "Treatment Adherence") OR "Adherence, Treatment") OR "Therapeutic Adherence") OR "Adherence, Therapeutic") OR "Lost to follow-up") OR "Lost to Follow Up") OR ("Therapeutic Adherence[MeSH Terms] AND Compliance"[MeSH Terms])) OR "Treatment Adherence"[MeSH Terms]) OR "Adherence, Treatment"[MeSH Terms]) OR "Therapeutic Adherence"[MeSH Terms]) OR "Adherence, Therapeutic"[MeSH Terms]) OR "Lost to Follow-Up"[MeSH Terms] | [149,280](https://www.ncbi.nlm.nih.gov/pmc/?cmd=HistorySearch&querykey=22) |
| ≠2 | (((((((((((((((("Dietary Supplements"[All Fields]) OR "Dietary counseling"[All Fields]) OR "Diet supplements"[All Fields]) OR "Food Supplementations"[All Fields]) OR "Food advice"[All Fields]) OR "Trace elements"[All Fields]) OR "nutritional supplements"[All Fields]) OR "nutritional advice"[All Fields]) OR "micronutrient supplementation"[All Fields]) OR "macronutrient supplementation"[All Fields]) OR minerals[All Fields]) OR vitamins[All Fields]) OR ("Zinc and Vitamin A supplementation"[All Fields])) OR "Vitamin D supplementation"[All Fields]) OR Dietary Supplements*[All Fields] OR *[MeSH Terms]) OR "Dietary Supplements[MeSH Terms]) OR minerals[MeSH Terms]) OR "[All Fields] AND trace elements[All Fields] AND "[MeSH Terms]) OR "[All Fields] OR Dietary Supplement[All Fields] OR "[MeSH Terms]) OR "[All Fields] OR Supplements, Dietary[All Fields] OR "[MeSH Terms]) OR "[All Fields] OR Dietary Supplementations[All Fields] OR "[MeSH Terms]) OR "[All Fields] OR Supplementations, Dietary[All Fields] OR "[MeSH Terms]) OR "[All Fields] OR Food Supplementations[All Fields] OR "[MeSH Terms]) OR "[All Fields] OR Food Supplements[All Fields] OR "[MeSH Terms]) OR "[All Fields] OR Food Supplement[All Fields] OR "[MeSH Terms]) OR "[All Fields] OR Supplement, Food MeSH Terms[All Fields]) | [256,891](https://www.ncbi.nlm.nih.gov/pmc/?cmd=HistorySearch&querykey=21) |
| ≠1 | ((((((((((((((((((Tuberculosis) OR TB) OR "Mycobacterium tuberculosis Infection") OR "Acid Fast Bacilli") OR AFB) OR "Tuberculosis, Multidrug-Resistant") OR MDR-TB) OR DR-TB) OR "Multidrug-Resistant Tuberculosis") OR "MDR Tuberculosis") OR "Tuberculosis, Drug Resistant") OR Tuberculosis[MeSH Terms]) OR "MDR Tuberculosis"[MeSH Terms]) OR TB[MeSH Terms]) OR "Tuberculosis, Pulmonary"[MeSH Terms]) OR "Mycobacterium tuberculosis Infection"[MeSH Terms]) OR "Multidrug-Resistant Tuberculosis"[MeSH Terms]) OR "Tuberculosis, Drug Resistant"[MeSH Terms]) | [473,007](https://www.ncbi.nlm.nih.gov/pmc/?cmd=HistorySearch&querykey=19) |

1. **Searching in Embase through Ovid**

| **No** | **Search terms** | **Results** |
| --- | --- | --- |
| ≠4 | ≠1 and ≠2 and ≠3 | 419 |
| ≠3 | Treatment adherence.mp. or patient compliance/ or patient compliance/ or Therapeutic Adherence.mp. or medication compliance/ or Lost to Follow Up.mp. or follow up/ | 2,138,295 |
| ≠2 | Dietary Supplements.mp. or dietary supplement/ or nutritional supplements.mp. or nutrition supplement/ or trace element/ or supplementation/ or micronutrient supplementation.mp. or diet supplementation/ or caloric intake/ or diet supplementation/ or macronutrient supplementation.mp. or retinol/ or macronutrient/ or vitamin D/ or vitamin supplementation/ or Vitamin D supplementation.mp. | 412,277 |
| ≠1 | Tuberculosis.mp. or lung tuberculosis/ or multidrug resistant tuberculosis/ or extensively drug resistant tuberculosis/ or Mycobacterium tuberculosis/ or tuberculosis/ or drug resistant tuberculosis/ | 343,582 |

1. **Searching in Scopus**

| **No** | **Search terms** | **Results** |
| --- | --- | --- |
| #4 | #1 AND #2 AND #3 | 389 document results |
| #3 | "Treatment adherence" OR "Therapeutic Adherence and Compliance" OR "Treatment Adherence" OR "Adherence, Treatment" OR "Therapeutic Adherence" OR "Adherence, Therapeutic" OR "Lost to follow-up" OR "Lost to Follow Up" OR "Therapeutic Adherence" OR "Therapeutic Compliance" OR "Treatment Adherence" | 70,373  document results |
| #2 | "Dietary Supplements" OR "Dietary counseling" OR "Diet supplements" OR "Food Supplementations" OR "Food advice" OR "Trace elements" OR "nutritional supplements" OR "nutritional advice" OR "micronutrient supplementation" OR "macronutrient supplementation" OR minerals OR vitamins OR "Zinc and Vitamin A supplementation" OR "Vitamin D supplementation" OR "Dietary Supplements*" OR "Dietary Supplements" OR minerals OR "trace elements" OR "Dietary Supplement" OR "Supplements, Dietary" OR "Dietary Supplementations" OR "Supplementations, Dietary" OR "Food Supplementations" OR "Food Supplements" OR "Food Supplement" OR "Supplement, Food" | 3,815,655  document results |
| ≠1 | "MDR Tuberculosis" OR "Tuberculosis, Drug Resistant" OR tuberculosis OR "MDR Tuberculosis" OR tb OR "Tuberculosis, Pulmonary" OR "Mycobacterium tuberculosis Infection" OR "Multidrug-Resistant Tuberculosis" OR "Tuberculosis, Drug Resistant" | 1,087,730document results |

1. **Searching in Web of Science**

| **No** | **Search terms** | **Results** |
| --- | --- | --- |
| 4 | #1 AND #2 AND #3 | 1046 |
| 3 | "Treatment Outcome" OR "Tuberculosis treatment outcome" OR "TB treatment outcome" OR "Cure rate" OR Recovery OR "Treatment completion" OR Mortality OR Mortalit* OR "Lost to follow-up" OR "Lost to Follow Up" OR "Treatment failure" OR "Treatment Failures" OR "Clinical response*" OR "Serum zinc" OR Zinc OR "Serum vitamin A" OR Retinol OR hemoglobin OR "hemoglobin status" | 2,937, 854 |
| 2 | "Dietary Supplements" OR "Dietary counseling" OR "Diet supplements" OR "Food Supplementations" OR "Food advice" OR "Trace elements" OR "nutritional supplements" OR "nutritional advice" OR "micronutrient supplementation" OR "macronutrient supplementation" OR minerals OR vitamins OR "Zinc and Vitamin A supplementation" OR "Vitamin D supplementation" OR "Dietary Supplements*" OR "Dietary Supplements" OR minerals OR "trace elements" OR "Dietary Supplement" OR "Supplements, Dietary" OR "Dietary Supplementations" OR "Supplementations, Dietary" OR "Food Supplementations" OR "Food Supplements" OR "Food Supplement" OR "Supplement, Food" | 992,891 |
| 1 | "MDR Tuberculosis" OR "Tuberculosis, Drug Resistant" OR tuberculosis OR "MDR Tuberculosis" OR tb OR "Tuberculosis, Pulmonary" OR "Mycobacterium tuberculosis Infection" OR "Multidrug-Resistant Tuberculosis" OR "Tuberculosis, Drug Resistant" | 396,096 |
