# Supplementary material for: Effectiveness of nutritional support to improve treatment adherence in patients with tuberculosis: a systematic review
Source: Nutr Rev. 2023 Sep 27;82(9):1216–25. doi: 10.1093/nutrit/nuad120 (PMC11317773; doi:10.1093/nutrit/nuad120)
Supplement: nuad120_Supplementary_Data [file nuad120_supplementary_data.zip › nuad120_Supplementary_Data/Supplementary_file_2_risk_of_bias_assessment_details.docx]

1. **Assessment of bias of RCTs included in the systematic review using RoB2**

| **Martins et al., 2009^S1^** | | |
| --- | --- | --- |
| **Risk of bias** | **Authors’ judgment** | **Support for judgement** |
| Random sequence generation (selection  bias) | **Low risk** | A research assistant randomly allocated study participants to the intervention group (supplementary food) or control group (nutritional advice). |
| Allocation concealment (selection bias) | **low risk** | The sequence was concealed from all investigators with sequentially numbered opaque sealed envelopes prepared distant from the study site. |
| Blinding of participants and personnel (performance bias) | **high risk** | Both participants and treatment providers were aware of an individual’s allocation status after randomization. |
| Blinding of outcome assessment (detection  bias) | **low risk** | An independent observer was blinded to the intervention received by the patients. |
| Incomplete outcome data (attrition bias)  All outcomes | **low risk** | Low proportion of loss to follow-up was observed in both groups (3% in the intervention vs 1% in the placebo groups). |
| Selective reporting (reporting bias) | **unclear risk** | There was no information about selective reporting. |
| Other bias | **low risk** | We were unable to identify any other  sources of bias. |
| **Benzekril et al., 2019^S2^** | | |
| **Risk of bias** | **Authors’ judgment** | **Support for judgment** |
| Random sequence generation (selection  bias) | **Low risk** | Participants were randomized to receive nutrition support or control groups. |
| Allocation concealment (selection bias) | **low risk** | Each subject blindly selected one card from the envelope to determine if they were randomized to intervention or control groups. |
| Blinding of participants and personnel (performance bias) | **low risk** | To decrease the possibility of stigmatization, both forms of nutrition support were provided in opaque containers to conceal the contents and were distributed. |
| Blinding of outcome assessment (detection  bias) | **Unclear risk** | Not documented in the original paper. |
| Incomplete outcome data (attrition bias)  All outcomes | **low risk** | There was no loss to follow-up in both groups. |
| Selective reporting (reporting bias) | **Low risk** | The authors reported results in the way they had originally proposed in the protocols |
| Other bias | **low risk** | We are unable to identify any other sources of  bias. |
| **Sudarsanam et al., 2011^S3^** | | |
| **Risk of bias** | **Authors’ judgement** | **Support for judgment** |
| Random sequence generation (selection  bias) | **low risk** | Participants were randomized to receive a nutritional supplement plus standard of care or standard of care alone by a computer-generated randomization code. |
| Allocation concealment (selection bias) | **Low risk** | Allocation was concealed; the randomization codes were in opaque envelopes opened by the dietician after dietary counseling was completed |
| Blinding of participants and personnel (performance bias) | **High risk** | There were no attempts made to blind any of the study team or participants. |
| Blinding of outcome assessment (detection  bias) | **Unclear** | There is no available evidence. |
| Incomplete outcome data (attrition bias)  All outcomes | **Low risk** | Low proportion of loss to follow-up was observed in both groups (3 out of 51in the intervention vs 3 out of 52 in the placebo groups). |
| Selective reporting (reporting bias) | **unclear risk** | It is unclear regarding selective reporting. |
| Other bias | **Low risk** | We are unable to detect any other source of bias. |
| **Jeremiah et al., 2014^S4^** | | |
| **Risk of bias** | **Authors’ judgment** | **Support for judgment** |
| Random sequence generation (selection  bias) | **Low risk** | Simple randomization, stratified by HIV status, was computed using the website to allocate patients to receive high-energy and vitamin/mineral-fortified biscuits as a nutritional supplement or no supplements. |
| Allocation concealment (selection bias) | **Low** | Eligible participants were randomized in a 1:1 allocation ratio. |
| Blinding of participants and personnel (performance bias) | **Unclear** | Placebo group subjects received organoleptically identical, subject codes remained sealed until after data analysis. |
| Blinding of outcome assessment (detection  bias) | **Unclear** | Placebo group subjects received organoleptically identical, Subject codes remained sealed until after data analysis” |
| Incomplete outcome data (attrition bias)  All outcomes | **low risk** | There was a low rate of attrition, <5% in both groups. |
| Selective reporting (reporting bias) | **unclear** | We could not retrieve the evidence about selective reporting. |
| Other bias | **low risk** | We did not identify any other sources of  bias. |

**References**

S1. Martins N, Morris P, Kelly PM. Food incentives to improve completion of tuberculosis treatment: randomised controlled trial in Dili, Timor-Leste. Bmj. 2009;339.

S2. Benzekri NA, Sambou JF, Tamba IT, et al. Nutrition support for HIV-TB co-infected adults in Senegal, West Africa: A randomized pilot implementation study. PLoS One. 2019;14(7):e0219118.

S3. Sudarsanam T, John J, Kang G, et al. Pilot randomized trial of nutritional supplementation in patients with tuberculosis and HIV–tuberculosis coinfection receiving directly observed short‐course chemotherapy for tuberculosis. Tropical Medicine & International Health. 2011;16(6):699-706.

S4. Jeremiah K, Denti P, Chigutsa E, et al. Nutritional supplementation increases rifampin exposure among tuberculosis patients coinfected with HIV. Antimicrobial agents and chemotherapy. 2014;58(6):3468-3474.

**2. Assessment of bias of non-randomized trials included in the systematic review using ROBINS-2.**

| **Filho C., 2009^S5^** | | |
| --- | --- | --- |
| **Risk of bias** | **Authors’ judgment** | **Support for judgment** |
| **Bias due to confounding** | **High** | Baseline and time-varying covariates were not handled |
| **Bias in the selection of participants** | **High** | Baseline and time-varying covariates were not handled |
| **Bias in classification of interventions** | **Low risk** | All patients ≥ 15 years of age and meet the criteria for a confirmed diagnosis of TB |
| **Bias due to deviations from intended interventions** | **unclear** | unclear |
| **Bias in measurement of outcomes** | **High risk** | The assessors were aware of interventions received by study participants. |
| **Bias due to missing data** | **Low risk** | Less than 5% of dropouts |
| **Bias in selection of the reported result** | **unclear** | We did not evaluate the reporting bias as the study protocols were not available |
| **Garden et al., 2013^S6^** | | |
| **Risk of bias** | **Authors’ judgment** | **Support for judgment** |
| **Bias due to confounding** | **High** | Baseline and time-varying covariates were not handled |
| **Bias in the selection of participants** | **High** | Baseline and time-varying covariates were not handled |
| **Bias in classification of interventions** | **Low risk** | The control group and intervention group consisted of consecutive homeless individuals. |
| **Bias due to deviations from intended interventions** | **unclear** | Not documented in the original study. |
| **Bias in measurement of outcomes** | **High** | The assessors were aware of interventions received by study participants. |
| **Bias due to missing data** | **unclear risk** | Not documented in the original study. |
| **Bias in selection of the reported result** | **unclear** | We did not evaluate the reporting bias as the study protocols were not available. |
| **Bock et al., 2001^S7^** | | |
| **Risk of bias** | **Authors’ judgment** | **Support for judgment** |
| **Bias due to confounding** | **High** | Baseline and time-varying covariates were not handled |
| **Bias in the selection of participants** | **High** | Not documented in the original study |
| **Bias in classification of interventions** | **Unclear** | Not documented in the original study |
| **Bias due to deviations from intended interventions** | **unclear** | No available information |
| **Bias in measurement of outcomes** | **High** | The assessors were aware of interventions received by study participants. |
| **Bias due to missing data** | **Unclear** | Not documented in the original study |
| **Bias in selection of the reported result** | **unclear** | We did not evaluate the reporting bias as the study protocols were not available |
| **Hu et al., 2021^S8^** | | |
| **Risk of bias** | **Authors’ judgment** | **Support for judgment** |
| **Bias due to confounding** | **Low** | Baseline and time-varying covariates were not handled |
| **Bias in the selection of participants** | **High** | “Not documented in the original study |
| **Bias in classification of interventions** | **low** | Not documented in the original study |
| **Bias due to deviations from intended interventions** | **unclear** | No available information |
| **Bias in measurement of outcomes** | **High** | The assessors were aware of interventions received by study participants. |
| **Bias due to missing data** | **Low** | No missing data was identified |
| **Bias in selection of the reported result** | **unclear** | We did not evaluate the reporting bias as the study protocols were not available |

**References**

S5. Cantalice Filho JP. Food baskets given to tuberculosis patients at a primary health care clinic in the city of Duque de Caxias, Brazil: effect on treatment outcomes. Jornal Brasileiro de Pneumologia. 2009;35:992-997.

S6. Garden B, Samarina A, Stavchanskaya I, et al. Food incentives improve adherence to tuberculosis drug treatment among homeless patients in Russia. Scandinavian journal of caring sciences. 2013;27(1):117-122.

S7. Bock N, Sales R, Rogers T, DeVoe B. A spoonful of sugar...: improving adherence to tuberculosis treatment using financial incentives [Notes from the Field. The International Journal of Tuberculosis and Lung Disease. 2001;5(1):96-98.

S8. Hu B, Ren G, Zhao L. Effect of Health Education Combined with Dietary Guidance on Nutritional Indicator, Immune Level, and Quality of Life of Patients with Pulmonary Tuberculosis. Computational and Mathematical Methods in Medicine. 2021;2021
