# Supplementary material for: Effectiveness of nutritional support to improve treatment adherence in patients with tuberculosis: a systematic review
Source: Nutr Rev. 2023 Sep 27;82(9):1216–25. doi: 10.1093/nutrit/nuad120 (PMC11317773; doi:10.1093/nutrit/nuad120)
Supplement: nuad120_Supplementary_Data [file nuad120_supplementary_data.zip › nuad120_Supplementary_Data/Table_S1_ROB.docx]

**Table S1: Quality and risk of bias assessments of RCTs included in the systematic review**

| **First author** | **Selection bias** | **Performance bias** | **Detection bias** | **Attrition bias** | **Reporting bias** | **Other bias** |
| --- | --- | --- | --- | --- | --- | --- |
| Martins et al., 2009^S1^ | Low | High | Low | Low | Unclear | Low |
| Benzekril et al., 2019^S2^ | Low | Low | Unclear | Low | Low | Low |
| Sudarsanam et al., 2011^S3^ | Low | High | Unclear | Low | Unclear | Low |
| Jeremiah et al., 2014^S4^ | Low | Unclear | Low | Low | Unclear | Low |

**References**

S1. Martins N, Morris P, Kelly PM. Food incentives to improve completion of tuberculosis treatment: randomised controlled trial in Dili, Timor-Leste. Bmj. 2009;339.

S2. Benzekri NA, Sambou JF, Tamba IT, et al. Nutrition support for HIV-TB co-infected adults in Senegal, West Africa: A randomized pilot implementation study. PLoS One. 2019;14(7):e0219118.

S3. Sudarsanam T, John J, Kang G, et al. Pilot randomized trial of nutritional supplementation in patients with tuberculosis and HIV–tuberculosis coinfection receiving directly observed short‐course chemotherapy for tuberculosis. Tropical Medicine & International Health. 2011;16(6):699-706.

S4. Jeremiah K, Denti P, Chigutsa E, et al. Nutritional supplementation increases rifampin exposure among tuberculosis patients coinfected with HIV. Antimicrobial agents and chemotherapy. 2014;58(6):3468-3474.
