# Supplementary material for: Effectiveness of nutritional support to improve treatment adherence in patients with tuberculosis: a systematic review
Source: Nutr Rev. 2023 Sep 27;82(9):1216–25. doi: 10.1093/nutrit/nuad120 (PMC11317773; doi:10.1093/nutrit/nuad120)
Supplement: nuad120_Supplementary_Data [file nuad120_supplementary_data.zip › nuad120_Supplementary_Data/Table_S2_ROB.docx]

**Table S2: Quality and risk of bias assessments of non-RCTs included in the systematic review**

| **First author** | **Confounding**  **Bias** | **Selection bias** | **Bias in the classification of interventions** | **Bias due to deviations**  **from intended**  **interventions** | **Measurement of outcome** | **Attrition bias** | **Reporting bias** |
| --- | --- | --- | --- | --- | --- | --- | --- |
| Filho C., 2009^S1^ | High | High | Low | Unclear | High | Low | Unclear |
| Garden et al., 2013^S2^ | High | High | Low | Unclear | High | Unclear | Unclear |
| Bock et al., 2001^S3^ | High | High | Unclear | Unclear | High | Unclear | Unclear |
| Hu et al., 2021^S4^ | Low | High | Low | Unclear | High | Low | Unclear |

**References**

S1. Cantalice Filho JP. Food baskets given to tuberculosis patients at a primary health care clinic in the city of Duque de Caxias, Brazil: effect on treatment outcomes. Jornal Brasileiro de Pneumologia. 2009;35:992-997.

S2. Garden B, Samarina A, Stavchanskaya I, et al. Food incentives improve adherence to tuberculosis drug treatment among homeless patients in Russia. Scandinavian journal of caring sciences. 2013;27(1):117-122.

S3. Bock N, Sales R, Rogers T, DeVoe B. A spoonful of sugar...: improving adherence to tuberculosis treatment using financial incentives [Notes from the Field. The International Journal of Tuberculosis and Lung Disease. 2001;5(1):96-98.

S4. Hu B, Ren G, Zhao L. Effect of Health Education Combined with Dietary Guidance on Nutritional Indicator, Immune Level, and Quality of Life of Patients with Pulmonary Tuberculosis. Computational and Mathematical Methods in Medicine. 2021;2021
